# Lipoprotein(a) is associated with coronary inflammation in people with HIV and undetectable HIV RNA

**DOI:** 10.1093/ehjimp/qyag040

**Published:** 2026-03-04

**Authors:** Nadim Nasrallah, Tarek Harb, Mark Atallah, Gary Gerstenblith, Sabina Haberlen, Theodoros Kelesidis, Jared W Magnani, Valentina Stosor, Kenneth Chan, Cheerag Shirodaria, Henry W West, Todd T Brown, Allison G Hays, Wendy S Post, Charalambos Antoniades, Thorsten M Leucker

**Affiliations:** Division of Cardiology, Department of Medicine, Johns Hopkins University School of Medicine, Halsted 500, Baltimore, MD 21287, USA; Division of Cardiology, Department of Medicine, Johns Hopkins University School of Medicine, Halsted 500, Baltimore, MD 21287, USA; Division of Cardiology, Department of Medicine, Johns Hopkins University School of Medicine, Halsted 500, Baltimore, MD 21287, USA; Division of Cardiology, Department of Medicine, Johns Hopkins University School of Medicine, Halsted 500, Baltimore, MD 21287, USA; Department of Epidemiology, Johns Hopkins Bloomberg School of Public Health, Baltimore, MD, USA; Department of Medicine, Division of Infectious Diseases, University of Texas Southwestern Medical Center, Dallas, TX, USA; Center for Research on Health Care, Department of Medicine, University of Pittsburgh, Pittsburgh, PA, USA; Divisions of Infectious Diseases and Organ Transplantation, Feinberg School of Medicine, Northwestern University, Chicago, IL, USA; Acute Multidisciplinary Imaging and Interventional Centre, Division of Cardiovascular Medicine, Radcliffe Department of Medicine, University of Oxford, Oxford, UK; Department of Cardiology, Oxford University Hospitals NHS Foundation Trust, Oxford, UK; Central Clinical School, Sydney Medical School, The University of Sydney, Sydney, New South Wales, Australia; Division of Endocrinology, Diabetes, and Metabolism, Department of Medicine, Johns Hopkins University, Baltimore, MD, USA; Division of Cardiology, Department of Medicine, Johns Hopkins University School of Medicine, Halsted 500, Baltimore, MD 21287, USA; Division of Cardiology, Department of Medicine, Johns Hopkins University School of Medicine, Halsted 500, Baltimore, MD 21287, USA; Department of Epidemiology, Johns Hopkins Bloomberg School of Public Health, Baltimore, MD, USA; Acute Multidisciplinary Imaging and Interventional Centre, Division of Cardiovascular Medicine, Radcliffe Department of Medicine, University of Oxford, Oxford, UK; Division of Cardiology, Department of Medicine, Johns Hopkins University School of Medicine, Halsted 500, Baltimore, MD 21287, USA

**Keywords:** lipoprotein(a), coronary inflammation, perivascular fat attenuation index, coronary computed tomography angiography, HIV

## Abstract

**Aims:**

People with HIV (PWH) and undetectable virus experience elevated cardiovascular risk independent of traditional risk factors. Vascular inflammation may contribute to this residual risk. The perivascular fat attenuation index (FAI), derived from coronary computed tomography angiography (CCTA), is a biomarker of coronary inflammation. Lipoprotein(a) [Lp(a)] carries oxidized phospholipids that may promote inflammation. Statins have demonstrated cardiovascular benefit in PWH, including pleiotropic anti-inflammatory effects. This study assessed the associations of Lp(a) and of statin use with coronary inflammation (FAI) in men with HIV (MWH).

**Methods and results:**

We analysed FAI of the left anterior descending (LAD) and the right coronary arteries (RCA) in 583 men from the Multicenter AIDS Cohort Study, a prospective, multicentre cohort study, including 280 with undetectable HIV RNA, <50 copies/ml. Associations between log_10_[Lp(a)] and LAD and RCA FAI were assessed using linear regression, adjusting for demographic and cardiovascular risk factors. Log_10_[Lp(a)] was associated with LAD FAI in MWH with undetectable HIV in adjusted analysis [+1.99 HU (0.38, 3.59); *P* = 0.02] but not among men without HIV (MWoH) or MWH with detectable HIV. Associations with RCA FAI were only significant in the unadjusted analysis. Statin use was associated with lower FAI, less inflammation in the LAD in MWH with undetectable virus, but did not modify the association between Lp(a) and coronary inflammation.

**Conclusion:**

Lp(a) was associated with increased coronary inflammation, independent of traditional cardiovascular risk factors, in MWH with undetectable virus. Statin therapy did not modify the relationship between coronary inflammation and Lp(a).

## Graphical Abstract

**Graphical Abstract:**
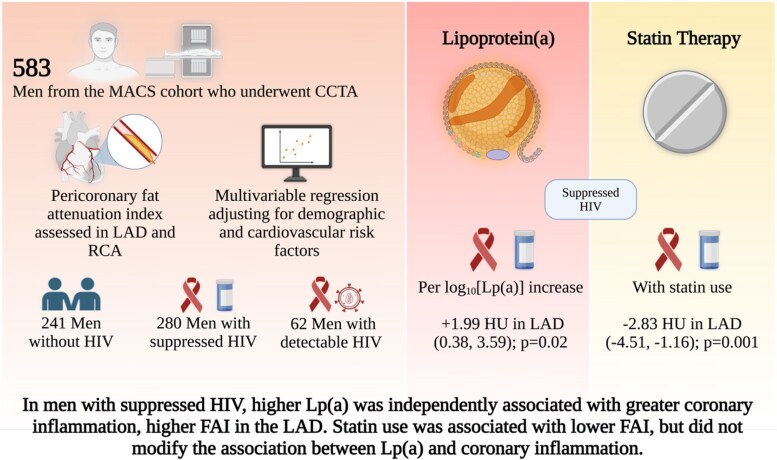

## Introduction

People with HIV (PWH) remain at elevated risk for cardiovascular disease (CVD) independent of traditional cardiovascular risk factors, despite effective antiretroviral therapy (ART) resulting in undetectable HIV RNA.^1,2^ Chronic immune activation, modulated by pro-inflammatory lipids,^3^ residual HIV replication in lymphoid tissues,^4^ and microbial translocation,^5^ may contribute to vascular inflammation and the progression of CVD.^1^ In the era of modern ART, HIV has transitioned to a chronic illness, and the prevalence of clinical and subclinical CVD among PWH is projected to increase to 78% by 2030.^6^ This underscores the importance of identifying factors contributing to residual risk in the clinically relevant, treated HIV population.

Imaging studies have provided evidence of subclinical coronary atherosclerosis and of coronary endothelial dysfunction in PWH.^7,8^ In the Multicenter AIDS Cohort Study (MACS), the prevalence and volume of non-calcified coronary artery plaque on coronary computed tomography angiography (CCTA) were greater in men with HIV (MWH) compared to men without HIV (MWoH).^7^ Perivascular fat attenuation index (FAI) is a validated, CT-derived biomarker that quantifies vascular inflammation by measuring perivascular adipose tissue attenuation on CCTA with Hounsfield units (HU), ranging from −190 to −30 HU.^9^ Less negative FAI values (closer to −30 HU), reflect increased inflammation and are independently associated with atherosclerotic cardiovascular disease, as well as cardiac and all-cause mortality.^9^

Lipoprotein (a) [Lp(a)] is elevated in PWH and is independently associated with CVD risk.^10^ It is also the major carrier of oxidized phospholipids, pro-inflammatory mediators that may contribute to this residual risk.^11^ The associations between continuous measures of Lp(a) and coronary inflammation assessed using FAI among people without HIV (PWoH) and PWH without, and those with, detectable virus in a large cohort were not previously reported.

Statin therapy significantly reduces cardiovascular events among PWH, possibly via both lipid-lowering and pleiotropic mechanisms.^12–15^ To date, the association of statin therapy with FAI in PWH, and whether statins modify any association between Lp(a) and FAI in PWoH and in PWH with undetectable virus have not been evaluated.

Leveraging MACS data,^7^ we measured CCTA-derived FAI and plasma Lp(a) concentrations to assess whether Lp(a) was associated with coronary inflammation in MWoH and in MWH with undetectable and those with detectable virus. We also investigated whether statin therapy modified any relationship between Lp(a) and FAI.

## Methods

### Study population

The MACS, now part of the Multicenter AIDS Cohort Study/Women’s Interagency HIV Study Combined Cohort Study (MWCCS), is an ongoing prospective, multicentre, cohort study evaluating the impact of HIV-1 infection on cardiovascular and other non-communicable disease outcomes among men with and without HIV who have sex with men in Baltimore, Chicago, Pittsburgh, and Los Angeles.^7^ Participants undergo semi-annual visits that include standardized clinical interviews, physical examinations, and laboratory assessments, with collection of blood and urine samples for biomarker analysis.

Eligible MACS participants were aged 40–70 years with a weight <300 lbs and no prior history of cardiac surgery or percutaneous coronary intervention, as these procedures would interfere with the measurement of the extent of coronary atherosclerosis. The scans were performed between January 2010 and August 2013. Institutional Review Board approval was obtained from all participating centres, and all participants provided written informed consent.

### CT scanning and FAI measurements

Cardiac CT was performed using 64- or 320- slice multidetector CT scanners following standard MACS imaging protocols, including heart rate control and administration of sublingual nitroglycerin prior to contrast injection.^7^ Scans were prospectively ECG-gated to minimize radiation exposure, and acquisition parameters were standardized across sites.^7^

Peri-coronary FAI was measured using CaRi-Heart algorithms.^9^ FAI was assessed in the fat surrounding the proximal 10 mm—50 mm segments of the left anterior descending (LAD) and right coronary (RCA) arteries. For the RCA, FAI was measured within a radial distance from the outer vessel wall equal to the vessel diameter. For the LAD, appropriate algorithms were applied to account for the variability of coronary anatomy and side branches.^16^ FAI was calculated as the weighted average attenuation of perivascular fat, ranging from −190 to −30 HU, adjusted for technical parameters, including tube voltage, contrast attenuation, and reconstruction kernel to account for inter-scan variability.^16^

### Lipoprotein(a) and clinical covariates

Lp(a) concentrations were measured using serum samples collected between 2010 and 2013. Samples were aliquoted at collection, stored at −80 °C, and did not undergo prior freeze-thaw cycles. Lp(a) concentrations were measured by Quest Diagnostics using an immunoturbidimetric assay standardized to World Health Organization and International Federation of Clinical Chemistry and Laboratory Medicine reference material.^17,18^ Results are reported in nmol/L.

Covariates were obtained from the MACS study visit closest to the CCTA scan, generally within 6 months, and included age, ethnicity, education, body mass index (BMI), systolic blood pressure, fasting lipid profile, fasting glucose, smoking status, and use of statin, antihypertensive, and antidiabetic medications. Diabetes mellitus was defined as a fasting serum glucose ≥126 mg/dl or use of glucose-lowering medications. Hypertension was defined as systolic blood pressure ≥130 mmHg, diastolic blood pressure ≥80 mmHg, or use of antihypertensive medications. HIV-related parameters included plasma HIV RNA (copies/mL) and ART duration. Undetectable HIV RNA was defined as <50 copies/mL.^19^

### Statistical analysis

Demographic and clinical characteristics are summarized by HIV serostatus and HIV viral suppression. Continuous variables are presented as means with standard deviations or medians with interquartile ranges, depending on distribution, and compared using a two-sample *t*-test or Wilcoxon rank-sum test. Non-parametric variables included in multivariate regression models were log-transformed. Categorical variables are reported as percentages and compared using the chi-square test. We stratified analyses into MWoH, MWH with undetectable RNA, and MWH with detectable viral RNA.

Multivariable linear regression was used to assess the association between log_10_[Lp(a)] and FAI. Analyses are restricted to participants with complete data on FAI, Lp(a), and all covariates specified in the fully adjusted model. Three models were constructed sequentially: model 1 is unadjusted; Model 2 adjusts for age, ethnicity (Black or non-Black ethnicity), education, and scanning centre; Model 3 is fully adjusted for age, ethnicity, education, scanning centre, BMI, systolic blood pressure, antihypertensive medications, total cholesterol, high density lipoprotein cholesterol (HDL-C), diabetes mellitus, current smoking, and statin use. We adjusted for scanning site to minimize variability in CT scanner models used across centres. Continuous variables were centred at the mean. Sensitivity analysis substituted low-density lipoprotein cholesterol (LDL-C) for total and HDL-C. To assess effect modification in MWoH and in MWH with undetectable HIV RNA, multiplicative interactions were tested between Lp(a) and HIV serostatus and between Lp(a) and statin use, adjusted for HIV serostatus, in fully adjusted models. Statistical analysis was performed using R version 4.4.1 (The R Foundation for Statistical Computing, Vienna, Austria).

## Results

### Demographics

Of the 583 men with complete data, 241 were MWoH and 342 were MWH. Among MWH, 280 had undetectable HIV RNA and 62 had detectable HIV RNA at the MACS visit closest to CT-imaging [median 51 days (21, 111)]. Mean LAD FAI was higher in the detectable than in the undetectable HIV group (−70.1 ± 7.6 vs. −72.6 ± 7.3 HU, *P* = 0.02, respectively), with a similar trend observed for RCA FAI (−70.5 ± 8.1 vs. −72.6 ± 8.2 HU; *P* = 0.06). Median Lp(a) levels trended higher among those with detectable than in those with undetectable HIV. Those with detectable HIV were more likely to be Black, and less likely to be on statin therapy or on ART compared to those with undetectable virus.

Compared to MWoH, MWH with undetectable HIV were younger, had lower BMI and HDL-C, and higher triglyceride levels. Median Lp(a) concentrations and mean FAI in the LAD and RCA FAI were similar among these two cohorts.

The full study cohort characteristics by HIV serostatus and HIV viral suppression are presented in *Table 1*.

**Table 1:** Full study cohort characteristics by HIV serostatus and HIV viral suppression

Variable	Men without HIV	Men with undetectable HIV	*P*-value*	Men with detectable HIV	*P*-value^†^
Sample size, *n*	241	280		62	
Age, years	55.5 ± 7.4	52.4 ± 6.6	**<0.001**	50.5 ± 6.3	**0**.**03**
Ethnicity					
White, *n* (%)	167 (69.3%)	160 (57.1%)	**<0**.**001**	18 (29.0%)	**<0**.**001**
Black, *n* (%)	58 (24.1%)	72 (25.7%)		36 (58.1%)	
Hispanic, *n* (%)	12 (5.0%)	47 (16.8%)		8 (12.9%)	
Asian, *n* (%)	2 (0.8%)	0 (0.0%)		0 (0.0%)	
Other, *n* (%)	2 (0.8%)	1 (0.4%)		0 (0.0%)	
Education					
No high school degree, *n* (%)	8 (3.3%)	21 (7.5%)	0.10	7 (11.3%)	0.14
High school degree, *n* (%)	83 (34.4%)	130 (46.4%)		36 (58.1%)	
College degree, *n* (%)	95 (39.4%)	88 (31.4%)		11 (17.7%)	
Postgraduate degree, *n* (%)	55 (22.8%)	41 (14.6%)		8 (12.9%)	
Tobacco use					
Never smoker, *n* (%)	66 (27.4%)	98 (35.0%)	0.06	13 (21.0%)	**0**.**002**
Former smoker, *n* (%)	122 (50.6%)	113 (40.4%)		20 (32.3%)	
Current smoker, *n* (%)	53 (22.0%)	69 (24.6%)		29 (46.8%)	
Smoking pack-years	2.5 [0.0, 21.5]	2.7 [0.0, 19.6]	0.97	8.1 [0.0, 20.1]	0.15
BMI, kg/m^2^	27.3 ± 4.7	26.0 ± 4.8	**0**.**002**	25.3 ± 4.1	0.24
Systolic blood pressure, mmHg	128.6 ± 14.7	126.3 ± 14.7	0.08	126.6 ± 15.4	0.79
Hypertension (%)	143 (59.3%)	157 (56.1%)	0.50	33 (53.2%)	0.79
Hypertension medication, *n* (%)	74 (30.7%)	85 (30.4%)	1.00	18 (29.0%)	0.96
Glucose, mg/dl	98.7 ± 27.4	102.6 ± 25.8	0.10	97.2 ± 18.4	0.06
Diabetes, *n* (%)	19 (7.9%)	33 (11.8%)	0.18	8 (12.9%)	0.98
Diabetes medications, *n* (%)	15 (6.2%)	25 (8.9%)	0.32	6 (9.7%)	1.00
Total cholesterol, mg/dl	194.7 ± 38.7	191.8 ± 38.2	0.38	176.3 ± 38.8	**0**.**005**
LDL cholesterol, mg/dl	117.0 ± 33.3	111.5 ± 35.3	0.07	101.4 ± 32.6	**0**.**03**
HDL cholesterol, mg/dl	53.1 ± 15.0	47.8 ± 13.9	**<0**.**001**	49.6 ± 18.4	0.49
Triglycerides, mg/dl	103.0 [74.0,146.0]	131.00 [94.8, 207.0]	**<0**.**001**	102.0 [86.3, 154.0]	**0**.**008**
Lipoprotein(a), nmol/l	26.0 [10.0, 78.0]	29.5 [9.0, 76.0]	0.71	39.5 [13.0, 80.8]	0.21
Statin use, *n* (%)	79 (32.8%)	108 (38.6%)	0.20	12 (19.4%)	**0**.**006**
Serum creatinine, mg/dl	1.0 ± 0.2	1.0 ± 0.2	0.25	1.0 ± 0.2	0.98
LAD FAI (HU)	−72.2 ± 7.3	−72.6 ± 7.3	0.57	−70.1 ± 7.6	**0**.**02**
RCA FAI (HU)	−72.2 ± 8.3	−72.6 ± 8.2	0.55	−70.5 ± 8.1	0.06
HIV clinical factors					
Viral load (copies/ml)				1232 [150, 15900]	
HAART duration (years)		9.5 (7.2, 12.7)		7.9 (1.3, 11.2)	<0.001
HAART regimen					
No HAART		12 (4.3%)		23 (37.1%)	<0.001
PI HAART		117 (41.8%)		30 (48.4%)	
NNRTI HAART		134 (47.9%)		6 (9.7%)	
NRTI HAART		2 (0.7%)		1 (1.6%)	
Other		15 (5.4%)		2 (3.2%)	

Data are reported as mean (standard deviation) or percentage. *P*-values are unadjusted. Median [interquartile range] for non-normally distributed variables and mean ± SD for normally distributed variables. HAART = highly active antiretroviral therapy. Bold *P*-values indicate statistical significance (*P* <0.05).

^*^ *P*-value comparing variables between seronegative and seropositive, HIV RNA <50 copies/ml.

^†^ *P*-value comparing variables between seropositive, HIV RNA <50 copies/ml, and HIV RNA ≥50 copies/ml.

### Lipoprotein(a) and coronary inflammation

**Figure 1:**
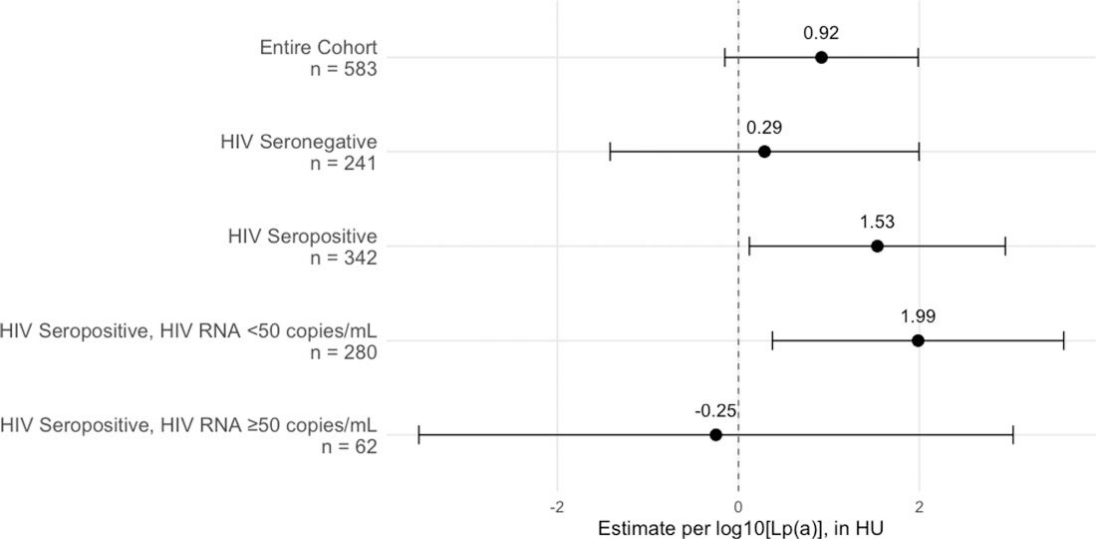
Forest plot showing fully adjusted estimates for the change in LAD FAI (HU) per 1-unit increase in log_10_[Lp(a)]. Models were adjusted for age, ethnicity, scanning centre, education, BMI, smoking status, systolic blood pressure, hypertension treatment, diabetes, statin use, total cholesterol, and HDL cholesterol. The overall model also adjusted for HIV serostatus.

The associations between Lp(a) and coronary inflammation in the LAD for the overall cohort and subgroups stratified by HIV serostatus and viral suppression using fully adjusted linear regression models are presented in *Figure 1*. In the entire cohort, higher log_10_[Lp(a)] trended towards higher LAD FAI [+0.92 HU (−0.15, 1.99); *P* = 0.09]. The association was statistically significant among MWH [+1.53 HU (0.12, 2.95); *P* = 0.03], but not among MWoH [+0.29 HU (−1.42, 2.00); *P* = 0.74]. After stratifying by HIV suppression status, the association between Lp(a) and coronary inflammation remained significant in MWH with undetectable virus [+1.99 HU (0.38, 3.59); *P* = 0.02], whereas no significant association was observed in the smaller cohort of those with detectable HIV RNA. In the RCA, the association between log_10_[Lp(a)] and FAI was significant in Model 1, but not after adjustment for covariates (Model 2 and Model 3). *Table 2* presents the associations between log_10_[Lp(a)] and FAI across the sequential statistical models.

**Table 2:** Multivariable linear regression models assessing the association between log_10_[Lp(a)] and FAI

Cohort	Model 1	Model 2	Model 3
LAD			
Entire cohort	2.47 (1.28, 3.66); ***P*** **<** **0.001**	0.88 (−0.24, 1.99); *P* = 0.12	0.92 (−0.15, 1.99); *P* = 0.09
Without HIV	1.65 (−0.19, 3.49); *P* = 0.08	0.76 (−0.97, 2.49); *P* = 0.39	0.29 (−1.42, 1.99); *P* = 0.74
With undetectable HIV RNA	3.43 (1.72, 5.13); ***P*** **<** **0.001**	1.83 (0.16, 3.49); ***P*** **=** **0.03**	1.99 (0.38, 3.59); ***P*** **=** **0.02**
With detectable HIV RNA	0.74 (−3.19, 4.67); *P* = 0.71	−1.04 (−4.53, 2.44); *P* = 0.55	−0.25 (−3.53, 3.03); *P* = 0.88
RCA			
Entire cohort	1.98 (0.63, 3.33); ***P*** **=** **0.004**	0.51 (−0.80, 1.81); *P* = 0.44	0.63 (−0.66, 1.92); *P* = 0.34
Without HIV	0.69 (−1.44, 2.81); *P* = 0.52	0.25 (−1.81, 2.31); *P* = 0.81	−0.37 (−2.43, 1.69); *P* = 0.72
With undetectable HIV RNA	2.68 (0.76, 4.61); ***P*** **=** **0.006**	0.91 (−1.02, 2.85); *P* = 0.36	1.24 (−0.68, 3.16); *P* = 0.21
With detectable HIV RNA	3.25 (−0.95, 7.46); *P* = 0.13	1.69 (−2.30, 5.67); *P* = 0.40	2.57 (−1.54, 6.69); *P* = 0.21

Sequential multivariable linear regression models assessing the association between log_10_[Lp(a)] and FAI in the LAD and RCA (HU) among men without HIV, with undetectable HIV RNA, and with detectable HIV RNA. Model 1 includes Lp(a) only. Model 2 adjusts for age, Black ethnicity, educational attainment, and centre. Model 3 additionally adjusts for BMI, statin therapy, current smoking status, systolic blood pressure, hypertension medications, diabetes, total cholesterol, and HDL cholesterol. Results are presented as the estimated absolute change in FAI (HU) per 1 log_10_-unit increase in Lp(a). Bold *P*-values indicate statistical significance (*P* < 0.05).

In the overall study cohort (*n* = 583, *Table 3*), HIV serostatus was not independently associated with LAD or RCA FAI. To evaluate whether the association between Lp(a) and coronary inflammation in the LAD differed between MWoH and MWH with undetectable HIV, an interaction term between log_10_[Lp(a)] and HIV in fully adjusted models was tested and was +1.18 HU (−1.02, +3.36); *P* = 0.29.

**Table 3:** Fully adjusted multivariable linear regression model assessing associations of covariates with LAD FAI in the entire cohort (*n* = 583)

Model 3				
	LAD		RCA	
Variable	Estimate (95% CI)	*P*-value	Estimate (95% CI)	*P*-value
Intercept	−72.53 (−75.10, −69.95)	**<0**.**001**	−73.76 (−76.87, −70.66)	**<0**.**001**
log_10_[Lp(a)]	0.92 (−0.15, 1.99)	0.09	0.63 (−0.66, 1.92)	0.34
HIV seropositive	−0.38 (−1.46, 0.71)	0.50	−0.96 (−2.27, 0.36)	0.15
Age	−0.00 (−0.08, 0.08)	0.98	−0.06 (−0.16, 0.04)	0.22
Black ethnicity	4.71 (3.36, 6.06)	**<0**.**001**	4.46 (2.84, 6.08)	**<0**.**001**
Educational attainment	0.32 (−0.08, 0.72)	0.11	0.17 (−0.30, 0.65)	0.48
Statin therapy	−2.74 (−3.88, −1.60)	**<0**.**001**	−1.47 (−2.84, −0.10)	**0**.**04**
Current smoking status	1.16 (−0.05, 2.38)	0.06	0.67 (−0.80, 2.14)	0.37
Systolic blood pressure	0.00 (−0.04, 0.04)	0.94	−0.01 (−0.05, 0.04)	0.72
Hypertension medications	−0.14 (−1.32, 1.04)	0.82	0.45 (−0.97, 1.87)	0.54
Diabetes	0.58 (−1.17, 2.32)	0.52	−0.12 (−2.26, 2.01)	0.91
Total cholesterol	0.00 (−0.02, 0.01)	0.79	−0.01 (−0.03, 0.00)	0.13
HDL cholesterol	0.03 (−0.01, 0.06)	0.14	0.04 (−0.00, 0.09)	0.07

Multivariable linear regression model assessing the association between log_10_[Lp(a)] and FAI in the LAD and RCA (HU) in men with and without HIV (*n* = 583). Model 3 adjusts for HIV status, age, black ethnicity, educational attainment, BMI, centre, statin therapy, current smoking, systolic blood pressure, hypertension medications, diabetes, total cholesterol, and HDL cholesterol. Results are presented as estimated absolute change in FAI in HU. Bold *P*-values indicate statistical significance (*P* < 0.05).

Multivariable analyses were conducted in MWoH (*n* = 241) and MWH with undetectable HIV RNA (*n* = 280) and the results are presented in *Tables 4* and *5*, respectively. In unadjusted models, log_10_[Lp(a)] was associated with higher LAD FAI only among MWH with undetectable HIV [+3.43 HU (1.73, 5.13); *P* < 0.001] and remained significant in Model 2. Lp(a) remained significantly associated with LAD FAI in the undetectable group in sensitivity analyses for Model 3 that replaced total and HDL-C with LDL-C [+1.77 HU (0.14, 3.41); *P* = 0.03] and additionally adjusting for current protease inhibitor therapy [+1.96 HU (0.35, 3.58); *P* = 0.017].

**Table 4:** Fully adjusted multivariable linear regression model assessing associations of covariates with LAD FAI in men without HIV (*n* = 241)

Model 3				
	LAD		RCA	
Variable	Estimate (95% CI)	*P*-value	Estimate (95% CI)	*P*-value
Intercept	−75.84 (−80.19, −71.50)	**<0**.**001**	−75.85 (−81.10, −70.60)	**<0**.**001**
log_10_[Lp(a)]	0.29 (−1.42, 1.99)	0.74	−0.37 (−2.43, 1.69)	0.72
Age	−0.01 (−0.13, 0.12)	0.92	−0.05 (−0.20, 0.10)	0.52
Black ethnicity	5.93 (3.58, 8.28)	**<0**.**001**	4.99 (2.17, 7.81)	**0**.**001**
Educational attainment	0.76 (0.07, 1.44)	**0**.**03**	0.53 (−0.28, 1.35)	0.20
Statin therapy	−2.84 (−4.71, −0.97)	**0**.**003**	−2.17 (−4.40, 0.06)	0.06
Current smoking status	1.15 (−1.01, 3.31)	0.29	1.16 (−1.44, 3.75)	0.38
Systolic blood pressure	−0.04 (−0.10, 0.02)	0.14	−0.07 (−0.14, 0.00)	0.06
Hypertension medications	−0.38 (−2.36, 1.61)	0.71	−0.53 (−2.91, 1.85)	0.66
Diabetes	−0.52 (−3.66, 2.63)	0.75	0.44 (−3.43, 4.30)	0.82
Total cholesterol	0.00 (−0.02, 0.03)	0.68	0.01 (−0.02, 0.03)	0.64
HDL cholesterol	0.04 (−0.02, 0.10)	0.21	0.01 (−0.06, 0.08)	0.77

Multivariable linear regression model assessing the association between log_10_[Lp(a)] and FAI in the LAD and RCA (HU) in men without HIV (*n* = 241). Model 3 adjusts for HIV status, age, Black ethnicity, educational attainment, BMI, centre, statin therapy, current smoking, systolic blood pressure, hypertension medications, diabetes, total cholesterol, and HDL cholesterol. Results are presented as estimated absolute change in FAI in HU. Bold *P*-values indicate statistical significance (*P* < 0.05).

**Table 5:** Fully adjusted multivariable linear regression model assessing associations of covariates with LAD FAI in men with HIV with undetectable HIV RNA (*n* = 280)

Model 3				
	LAD		RCA	
Variable	Estimate (95% CI)	*P*-value	Estimate (95% CI)	*P*-value
Intercept	−70.81 (−74.20, −67.41)	**<0**.**001**	−74.06 (−78.10, −70.02)	**<0**.**001**
log_10_[Lp(a)]	1.99 (0.38, 3.59)	**0**.**02**	1.24 (−0.68, 3.16)	0.21
Age	0.03 (−0.10, 0.15)	0.65	−0.04 (−0.19, 0.11)	0.61
Black ethnicity	3.40 (1.38, 5.41)	**0**.**001**	3.90 (1.49, 6.31)	**0**.**002**
Educational attainment	0.11 (−0.45, 0.67)	0.70	−0.03 (−0.69, 0.64)	0.94
Statin therapy	−2.83 (−4.51, −1.16)	**0**.**001**	−0.87 (−2.87, 1.14)	0.40
Current smoking status	1.37 (−0.41, 3.14)	0.13	−0.15 (−2.28, 1.98)	0.89
Systolic blood pressure	0.03 (−0.03, 0.08)	0.39	0.02 (−0.04, 0.09)	0.49
Hypertension medications	−0.52 (−2.25, 1.20)	0.55	0.92 (−1.14, 2.99)	0.38
Diabetes	1.70 (−0.78, 4.19)	0.18	−0.53 (−3.56, 2.51)	0.73
Total cholesterol	0.00 (−0.02, 0.02)	0.95	−0.02 (−0.05, 0.00)	0.07
HDL cholesterol	0.02 (−0.04, 0.08)	0.46	0.08 (0.01, 0.15)	**0**.**02**

Multivariable linear regression model assessing the association between log_10_[Lp(a)] and FAI in the LAD and RCA (HU) in men with undetectable HIV (*n* = 280). Model 3 adjusts for HIV status, age, Black ethnicity, educational attainment, BMI, centre, statin therapy, current smoking, systolic blood pressure, hypertension medications, diabetes, total cholesterol, and HDL cholesterol. Results are presented as estimated absolute change in FAI in HU. Bold *P*-values indicate statistical significance (*P* < 0.05).

In fully adjusted models, Black ethnicity was independently associated with higher LAD FAI in both MWoH and MWH with undetectable HIV RNA. Positive nonsignificant associations were present for current smoking in both groups and for diabetes in the undetectable HIV group. No significant associations were observed for total cholesterol, HDL-C, systolic blood pressure, or hypertension medication use in either group.

The multivariable model intercept, which represents predicted LAD FAI for a reference individual [White, non-smoking male with mean values of Lp(a), cholesterol, and systolic blood pressure], was −75.8 HU in MWoH and −70.8 HU in MWH with undetectable HIV RNA, suggesting higher predicted LAD FAI in the latter group. Although this difference approached statistical significance based on a Wald test from the multivariable regression model (z = −1.79; *P* = 0.07), HIV serostatus was not independently associated with LAD or RCA FAI in the overall cohort. There was no significant difference in LAD FAI intercepts between MWoH and MWH with detectable HIV RNA, and no differences were observed across groups for RCA FAI.

### Statin therapy

**Figure 2:**
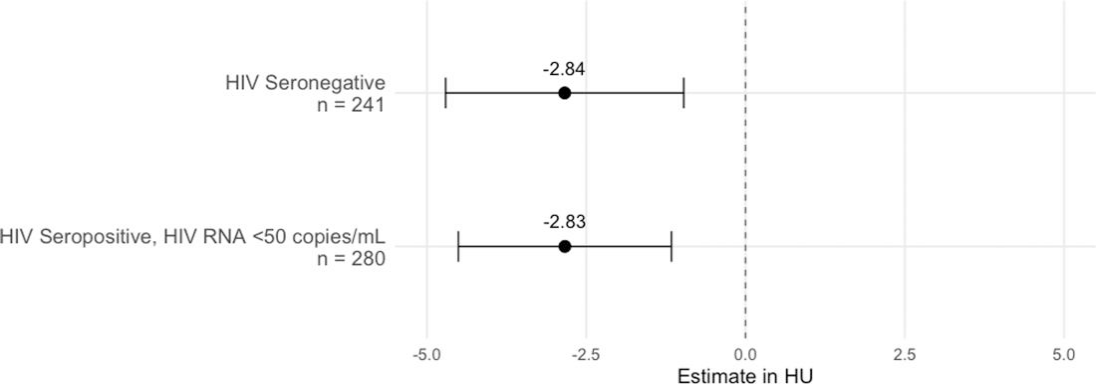
Association between statin use and LAD FAI (HU) in MWoH and MWH and undetectable HIV RNA.

In fully adjusted models, statin use was independently associated with a more negative LAD FAI in both MWH with undetectable RNA and MWoH, indicative of less inflammation (*Figure 2*). Among those with undetectable virus, statin therapy was associated with −2.83 HU (−4.51, −1.16); *P* < 0.001 lower LAD FAI. A similar association was observed in MWoH [−2.84 HU (−4.71, −0.97); *P* = 0.003]. These associations remained robust in sensitivity analyses replacing HDL-C and total cholesterol with LDL-C, with statin use still significantly associated with lower LAD FAI in both groups [undetectable MWH: −2.62 HU (−4.30, −0.95); *P* = 0.002; MWoH: −2.73 HU (−4.61, −0.85); *P* = 0.005].

In the RCA, there was a borderline non-significant trend between statin use and lower FAI among MWoH, with an estimated difference of −2.17 HU (−4.40, 0.06); *P* = 0.06). No significant association was observed between MWH and undetectable HIV RNA [−0.87 HU (−2.87, 1.14); *P* = 0.40].

**Figure 3:**
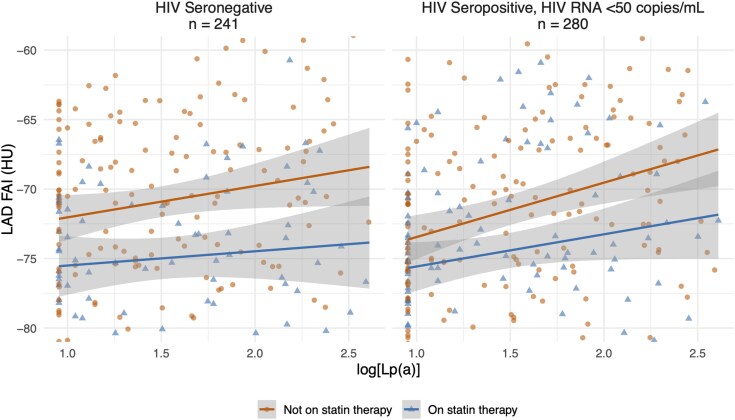
Scatter plot with fitted regression lines illustrating the association between log_10_[Lp(a)] and LAD FAI (HU), stratified by statin therapy and HIV serostatus. Shaded areas represent 95% confidence intervals. The left panel includes MWoH (*n* = 241), and the right panel includes MWH with undetectable HIV (<50 copies/mL; *n* = 280).

To evaluate whether the relationship between Lp(a) and coronary inflammation was modified by statin therapy, we included an interaction term between log_10_[Lp(a)] and statin use in a combined model of MWoH and undetectable HIV participants, adjusting for HIV status. The interaction was not statistically significant [+0.24 HU (−1.99, 2.47) ; *P* = 0.83], suggesting that the association between Lp(a) and LAD FAI did not differ by statin use (*Figure 3*).

## Discussion

We observed, for the first time, that Lp(a) levels were associated with coronary inflammation as measured by perivascular FAI among MWH with undetectable HIV in unadjusted and adjusted models, whereas no association was observed among MWoH. FAI is a CT-derived biomarker of coronary inflammation that predicts cardiac risk.^20^ These findings suggest that Lp(a) may contribute to cardiovascular risk in the context of undetectable HIV, even after controlling for traditional CVD risk factors. This relationship was absent in MWH with detectable HIV RNA.

PWH experience a 50–200% higher risk of myocardial infarction than do PWoH, independent of traditional CVD risk factors.^7,21–23^ Chronic inflammation and immune activation are believed to play a key role in this residual risk.^24–26^ Lp(a) has proinflammatory properties, notably as the primary carrier of oxidized phospholipids, which can trigger monocyte recruitment, resulting in arterial inflammation.^27^ Although Lp(a) is an independent risk factor for CVD in the general population and prior studies in PWoH demonstrated a link between Lp(a) and coronary inflammation,^28^ despite a small sample size, we did not observe a significant relationship between Lp(a) and FAI in our MWoH cohort.

Prior reports indicate that the +1.99 HU LAD FAI increment per log_10_[Lp(a)] in MWH with undetectable HIV is associated with differences in the degrees of coronary stenosis and presence of high-risk plaque phenotypes. Mátyás *et al*. reported that patients with severe stenosis of 70–99% in one or two coronary arteries (CAD-RADS 4A) had perivascular FAI values approximately 4 HU higher than those with less extensive disease.^29^ Similarly, Guo *et al*. reported mean FAI values approximately 4 HU higher in vulnerable compared to non-vulnerable plaques, defined by the presence of high-risk CT features such as low-attenuation plaque, positive remodelling, or spotty calcification.^30^ These studies suggest that the Lp(a)-associated increase in FAI observed in our cohort reflects a biologically meaningful degree of vascular inflammation, consistent with the understanding of Lp(a) as a contributor to residual atherosclerotic risk independent of other atherosclerotic risk factors. This relationship was absent in the RCA FAI after multivariable adjustment. RCA FAI assessment may be more susceptible to artefact due to proximity to the aortic wall, which can increase measurement noise.^31^ Moreover, the direction of effect for RCA FAI was consistent with that observed in the LAD but with wider confidence intervals, supporting the possibility of greater technical variability. In addition, the atherosclerotic burden in the LAD is generally more than that in the RCA, which may render LAD FAI a more sensitive marker of systemic inflammatory signalling.^32^

Zisman et al. examined coronary FAI, serum Lp(a) levels, soluble inflammatory markers, and activated monocyte and T-cell subsets in a single-centre study of 21 PWoH and 58 PWH with undetectable HIV.^25^ Lp(a) levels were divided into tertiles. Although there was no significant association between Lp(a) tertile and mean FAI in the LAD and RCA, the interaction term between Lp(a) tertile and HIV serostatus indicated a stronger association between Lp(a) and FAI in PWH and undetectable virus than in PWoH.^25^ Additionally, Lp(a) levels were correlated with soluble inflammatory markers and activated monocyte and T-cell subsets.^25^

In our multicentre cohort of 583 men, we used isoform-insensitive measurements and continuous, log-transformed Lp(a) data, with adjustment for a broad set of cardiovascular risk factors, including total and HDL-C, diabetes, and hypertension, and observed a consistent positive association between Lp(a) and LAD FAI in undetectable MWH. Statin therapy, while associated with lower LAD FAI, did not modify the relationship between Lp(a) and coronary inflammation. The importance of Lp(a) in promoting coronary vascular dysfunction is further supported by studies of PWH treated with PCSK9 inhibitors, which demonstrated that decreases in Lp(a), but not LDL-C, were associated with improvements in coronary endothelial function as assessed by magnetic resonance imaging.^33^ These observations suggest that Lp(a) may potentiate the inflammatory vascular effects of chronic HIV-related immune activation in MWH with undetectable virus.

Although HIV serostatus was not independently associated with LAD FAI, the estimated intercept in the undetectable HIV group (−70.8 HU) was elevated compared to the intercept for those without HIV (−75.8 HU, *P* = 0.07), suggesting a higher baseline level of coronary inflammation. Notably, the HIV group’s intercept approached the CRISP-CT study threshold of −70.1 HU, with individuals with less negative values having a 5.6-to-9.0-fold higher risk of cardiac death over a median follow-up of 72 months.^9^

In our cohort, Lp(a) was not associated with FAI among the 62 men with detectable HIV. This may reflect the significant impact of virus-driven inflammation in viremic MWH, potentially outweighing the contributions of other proinflammatory factors, such as Lp(a). HIV viremia is associated with systemic inflammation and endothelial cell dysfunction.^26^ The small sample size, limited statistical power, and lower ART and statin use in the viremic group may have also limited the ability to detect an association in this group. Larger studies including more individuals with detectable HIV RNA will be important to clarify the relationship between Lp(a) and coronary inflammation in this population.

Black ethnicity was consistently associated with higher FAI across our cohort, even after adjusting for traditional cardiovascular risk factors. This is consistent with prior studies reporting higher pericoronary fat attenuation among Black participants without HIV.^34^ Population studies show higher levels of inflammatory biomarkers (e.g. hsCRP and IL-6) and more impaired nitric oxide-mediated endothelial function in Black individuals, consistent with a higher perivascular inflammatory milieu.^35,36^ Beyond inflammation, the difference in FAI may reflect surrounding tissue composition and paracrine interactions. In addition, there is more brown adipose tissue, which has a higher water content and is more metabolically active than white adipose tissue, in Black individuals, both of which may increase attenuation.^37,38^ However, the relative contribution of these mechanisms to racial differences in FAI remains uncertain.

In our study, statin therapy was independently associated with lower coronary inflammation in both, MWoH and MWH with undetectable virus, after adjustment for total cholesterol and HDL-C, and for LDL-C, suggesting that the observed effect was not solely attributable to changes in lipid levels alone. The REPRIEVE trial demonstrated pleiotropic effects of statins beyond LDL-C lowering in PWH, including reductions in biomarkers of lipid oxidation (oxidized LDL-C) and arterial inflammation (lipoprotein-associated phospholipase A2), but not systemic inflammatory markers such as hsCRP and IL-6.^12,13^ Our findings provide novel evidence that statin therapy is associated with lower coronary inflammation in MWH, likely through both lipid lowering and anti-inflammatory pathways.

Despite its anti-inflammatory effects,^39^ statin therapy did not modify the relationship between Lp(a) and coronary inflammation in our cohort. It is known that statins have minimal impact on Lp(a) concentrations and may even cause a slight increase,^40^ though this does not appear to confer additional risk.^41^ In our analysis, no significant interaction was observed between statin use and Lp(a) levels on FAI, suggesting that the pro-inflammatory effect of Lp(a) persists in MWH despite statin therapy. This supports Lp(a) as a residual risk factor not addressed by standard lipid lowering therapies.

### Limitations

Our study population is limited to men, and our findings might not be generalizable to women with and without HIV. We measured Lp(a) at the same time rather than prior to the FAI outcome, though concerns of reverse-causation are not a large threat given that Lp(a) levels are largely genetically determined and stable.^18^ Our exposure of interest, Lp(a) levels, cannot be randomized, and while our observational study design is appropriate, we cannot exclude the possibility of residual confounding from other factors associated with both Lp(a) and FAI, such as duration of HIV infection, systemic inflammatory burden, and historical exposure to older ART drugs with known cardiometabolic effects. Additionally, there were differences in some cardiovascular risk factors, behaviours, and sociodemographic factors between our HIV subgroups, particularly among the small group of men with HIV viremia and differences in the Lp(a)-FAI association between groups may be affected by residual confounding. CT measurements of FAI were performed using early CaRi-Heart prototype algorithms, which may have introduced variability. While our cohort was substantially larger than prior studies, it may still be underpowered to detect modest interaction effects.

Moreover, CCTA involves exposure to ionizing radiation. Although more modern protocols use dose-reduction strategies, the radiation burden must be considered when evaluating imaging-derived inflammatory markers. Given the observational design and limited generalizability of the current study, routine clinical use of CCTA solely for pericoronary fat attenuation assessment cannot be recommended at this stage.

Nonetheless, this population-based study leverages a large, well-characterized cohort with standardized coronary CT imaging. Both groups were drawn from the same study population, enhancing internal validity and minimizing selection bias.

## Conclusions

Lp(a) was significantly associated with coronary inflammation among MWH with undetectable virus and therefore potentially contributes to the excess coronary disease risk observed despite control of viremia and traditional cardiovascular risk factors.^1^ Statin therapy was associated with lower coronary inflammation but did not mitigate the pro-inflammatory effect of elevated Lp(a), highlighting a gap in current treatment strategies. Routine measurement of Lp(a) in MWH may identify high-risk individuals who could benefit from more intensive preventive measures. Emerging Lp(a) lowering therapies, if proven to reduce cardiovascular events, may offer particular benefit in high-risk populations, however, dedicated trials are needed to establish their safety and efficacy in MWH.

## Lead author biography

Nadim Nasrallah holds a medical degree from the American University of Beirut, Lebanon. Soon after he graduated, he joined Johns Hopkins to work as a postdoctoral research fellow in the Division of Cardiology. He works on projects related to lipoprotein(a), vascular inflammation, and coronary endothelial function.
